# Modified Sensory Testing in Non-verbal Patients Receiving Novel Intrathecal Therapies for Neurological Disorders

**DOI:** 10.3389/fneur.2022.664710

**Published:** 2022-02-10

**Authors:** Laura Cornelissen, Carolina Donado, Timothy W. Yu, Charles B. Berde

**Affiliations:** ^1^Department of Anesthesiology, Critical Care and Pain Medicine, Boston Children's Hospital, Boston, MA, United States; ^2^Department of Anaesthesia, Harvard Medical School, Boston, MA, United States; ^3^Divisions of Genetics and Genomics, Department of Pediatrics, Boston Children's Hospital, Boston, MA, United States; ^4^Department of Paediatrics, Harvard Medical School, Boston, MA, United States

**Keywords:** pain measurement, antisense oligonucleotides, neurodevelopmental disorders, clinical trials, quantitative sensory testing, patient safety, personalized medicine, intrathecal

## Abstract

Several neurological disorders may be amenable to treatment with gene-targeting therapies such as antisense oligonucleotides (ASOs) or viral vector-based gene therapy. The US FDA has approved several of these treatments; many others are in clinical trials. Preclinical toxicity studies of ASO candidates have identified dose-dependent neurotoxicity patterns. These include degeneration of dorsal root ganglia, the cell bodies of peripheral sensory neurons. Quantitative sensory testing (QST) refers to a series of standardized mechanical and/or thermal measures that complement clinical neurologic examination in detecting sensory dysfunction. QST primarily relies on patient self-report or task performance (i.e., button-pushing). This brief report illustrates individualized pragmatic approaches to QST in non-verbal subjects receiving early phase investigational intrathecal drug therapies as a component of clinical trial safety protocols. Three children with neurodevelopmental disorders that include *Neuronal Ceroid Lipofuscinosis Type 7, Ataxia-Telangiectasia*, and *Epilepsy of Infancy with Migrating Focal Seizures* are presented. These case studies discuss individualized testing protocols, accounting for disease presentation, cognitive and motor function. We outline specific considerations for developing assessments for detecting changes in sensory processing in diverse patient groups and safety monitoring trials of early phase investigational intrathecal drug therapies. QST may complement information obtained from the standard neurologic examination, electrophysiologic studies, skin biopsies, and imaging. QST has limitations and challenges, especially in non-verbal subjects, as shown in the three cases discussed in this report. Future directions call for collaborative efforts to generate sensory datasets and share data registries in the pediatric neurology field.

## Introduction

Precision medicine is an emerging area for disease prevention and treatment strategies that takes into account individual variability in genes and environment. A growing number of neurological disorders may be amenable to precision medicine using treatment with gene-targeting therapies such as antisense oligonucleotides (ASOs) or viral vector-based gene therapy. The US Food and Drug Administration (FDA) and European Medicines Agencies (EMA) have approved several of these treatments for clinical applications; many others are in clinical trials.

Recent advances in ASO drug development have resulted in a rapid transition from bench-to-bedside candidates with the FDA recently approving 12 ASO therapies between the years 2016 and 2020 alone (1). All of these therapies were optimized for treatment of orphan diseases where druggable genetic targets are present only in small-to-moderate proportions of patients. Preclinical toxicity studies of ASO candidates in rodents and non-human primates have identified dose-dependent neurotoxicity patterns. These include acute and subacute dysfunction, inflammation, and degeneration of dorsal root ganglia, the cell bodies of peripheral sensory neurons (2, 3). It is critical to ensure these therapies are safe and effective in order to translate therapies into the clinic.

Clinicians are faced with challenges in identification and measurement of drug-related neurotoxicity in patients with neurological disorders using non-invasive bedside approaches. Quantitative sensory testing (QST) refers to a series of standardized measures that complement clinical neurologic examination in detecting sensory dysfunction (4–7). QST primarily relies on patient self-report or task performance (i.e., button-pushing). Little has been described of how best to design clinical trial protocols that accommodate patients with neurological disorders who are unable to provide self-report or have reduced functional capacity. This brief report illustrates individualized pragmatic approaches to QST in non-verbal subjects undergoing treatment with novel ASOs as a component of clinical trial safety protocols.

### Pharmacovigilance in Clinical Trials

Monitoring patient safety and drug toxicity during clinical trials is a critical component in drug development. Optimal safety biomarkers are quantitative and permit within-subject comparisons and group comparisons relative to age-specific norms. Developing broadly applicable safety biomarkers is challenging for patients with rare neurologic disorders. Sample sizes are small (or even N-of-1), and there is heterogeneity among different neurologic disorders or even participants with the same condition.

### Quantitative Sensory Testing

Hyposensitivity (elevated sensory thresholds) occurs with sensory loss, due to injury or disease (8, 9) or temporarily by local anesthesia (10, 11) or analgesia (12, 13). Hypersensitivity (lowered sensory thresholds) can occur with many types of neuropathic pain (5, 14, 15).

QST provides insight into large myelinated Aδ and small unmyelinated C fiber function (16). Typically, a calibrated stimulus is applied to the skin in graded intensities, and the patient's perception is recorded. Stimulus modalities can be mechanical (i.e., monofilaments, pin-prick, vibratory), thermal (i.e., Peltier thermode), or electrical (17, 18). Outcome measures include stimulus detection (present/absent) and stimulus intensity (self-reported rating).

Adult QST protocols are standardized and studied extensively. A widely used protocol involves a battery of 13 non-invasive assessments, developed by the German Neuropathic Pain network (DFNS) (5). A DFNS-QST profile of two body areas can be obtained within 1-h in adults with neuropathic pain (4).

Standardized protocols permit comparison of one individual to normative values and patients with neuropathic conditions using multicenter databases (5, 6, 14, 19–23). Patient stratification according to QST profile has been proposed to identify responders in analgesic trials (24). QST has been used in over 1,000 patients with malignancies to investigate large and small fiber neuropathies associated with chemotherapy agents, including bortezomib, vincristine, taxanes, and platinum compounds (25–28).

### QST Protocols in Children

Modifications to the DFNS protocol have been made for typically developing children as young as 6 years old. Reference values have been reported for age and gender (19, 22). Our research group has extensive experience in sensory profiling. We have developed QST protocols for typically-developing children (29), children and adolescents with juvenile idiopathic arthritis (30), complex regional pain syndrome (31–33), and scoliosis (34); and adult volunteers receiving novel local anesthetics in Phase-1 clinical trials (10).

For term and preterm neonates, infants, and non-verbal children, surrogate approaches to sensory evaluation have recorded stimulus-evoked behaviors (i.e., facial expressions or nocifensive leg flexion withdrawal response following cutaneous mechanical stimulation) (35–38). We recently examined longitudinal trends in nocifensive withdrawal responses among typically developing infants and infants undergoing prolonged intensive care for treatment of esophageal atresia (36).

Limited information has been reported regarding QST protocol design in non-verbal children. From PubMed search, we could not identify publications describing protocols for sensory assessments during drug trials for children with communicative, cognitive, and motor constraints.

### Case Examples

The examples below illustrate *individualized* testing protocols, accounting for disease presentation, cognitive and motor function for three children receiving spinal administration of a personalized ASO in an N-of-1 protocol (3). These patients have medically complex congenital neurodevelopmental disorders that present with varying degrees of neurodegeneration and marked functional impairment. To provide the reader with context, children with medical complexity often require substantial medical care, specialized therapy and educational support, and have varying severity of functional limitations that require tracheostomy, feeding tube or a wheelchair (39, 40).

The importance of monitoring sensory function and performing QST in these patients was motivated in part by preclinical spinal toxicology studies. These preclinical studies showed a safe behavioral and neuropathologic profile in the dose ranges chosen (based on scaling models) for clinical administration (3, 41, 42). At higher doses, animals showed varying degrees of dorsal root ganglion inflammation and nerve injury (3). For all cases, QST was performed to identify potential signs of drug-associated nerve injury, i.e., loss of response to tactile stimulation as a result of nerve damage; the results of QST remained stable throughout treatment.

#### Methods Overview

Lower extremities were tested because animal studies show more behavioral and histologic signs of toxicity around lumbar than thoracic or cervical spinal levels. Baseline assessments before treatment, and repeated tests pre- and post-drug administration were performed. The duration of treatments ranged from 12 to 36 months.

The primary outcome measure was the lowest mechanical stimulus intensity required to evoke nocifensive behavior (Case 1; Case 3) or verbal and non-verbal cues (Case 2) when applied to the foot. In all cases caregivers were present during the assessments and they engaged with the physiologist in order to help relax the patient or providing feedback about responses as needed.

All studies were conducted with local ethics approval and written informed parent consent.

## 6-Year-Old Girl with Neuronal Ceroid Lipofuscinosis Type 7

Patient 1 had typical neurodevelopment through infancy. By age 3, she began to develop a progressive decline of cognitive and motor function, blindness, seizures, and spasticity. At age 6, whole genome sequencing led to a diagnosis of neuronal ceroid lipofuscinosis Type 7 (3). At evaluation, the patient was non-verbal with significant developmental delay and was unable to walk unassisted. Testing with the use of Vineland Adaptive Behavior Scales (Vineland-II) showed low scores for Communication, Daily Living Skills, Socialization and Motor Skill domains, with declines in 7 of 11 neurologic and neuropsychological subscores at baseline. Gross Motor Function Measure-88 scores were 0 on the “Crawling and Kneeling,” “Standing,” and “Walking, Running and Jumping” domains at baseline.

**Figure 1:**
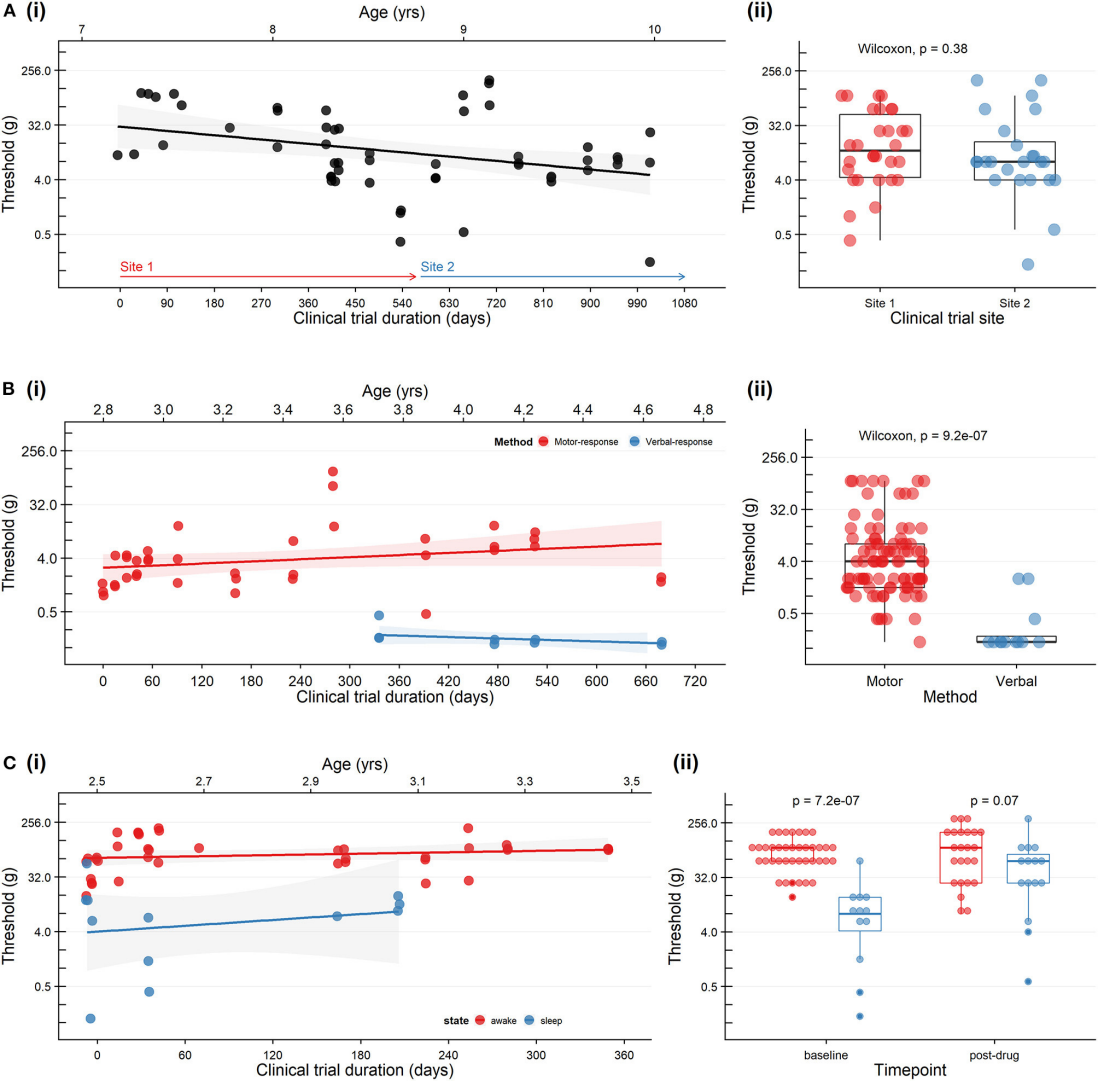
Trends in cutaneous mechanical threshold in three patients with neurodegenerative disorders receiving a personalized investigational intrathecal ASO. **(A)** *A patient with Neuronal Ceroid Lipofuscinosis Type 7 who was studied at two study sites*. **(i)** Baseline sensory thresholds recorded on the day of drug dosing (prior to drug administration). Study site 1 (red arrow) was relocated to a second site (blue arrow) on Day 603. **(ii)** Baseline sensory threshold data collected at site 1 and site 2 (by site-specific sensory physiologists) showing no clinical or statistically significant differences (Wilcoxon). **(B)** *A patient with Ataxia-Telangiectasia during developmental progression from preverbal to verbal status*. Leg flexion withdrawal or feet movement was used consistently over 10 months of the study (motor response: red). At age 3.8 years the patient was able to provide verbal responses as the primary outcome measure (verbal response: blue) and therefore we modifed the protocol to improve engagement and responses. **(i)** Baseline sensory thresholds recorded on the day of drug dosing (prior to drug administration). **(ii)** Differences between sensory threshold with verbal and motor responses (Wilcoxon). **(C)** *A patient with Epilepsy of Infancy with Migrating Focal Seizures who exhibited considerable state-dependent variability in the sensory threshold*. **(i)** Baseline sensory thresholds were recorded on the day of drug administration (prior to drug administration) (wakefulness: red; sleep: blue). **(ii)** Sensory threshold data showing lower sensitivity to mechanical stimulation during wakefulness (red) compared to sleep (blue) at baseline (Wilcoxon). Pre-dose *and* post-dose sensory thresholds are included in the plot; no statistical difference in pre-drug vs. post-drug sensory thresholds (Wilcoxon, *p* = 0.53). *Sensory thresholds were established by applying von Frey hair monofilaments to the plantar surface of the foot with increasing stimulus intensity. For all plots: each dot represents an individual trial; the scatter plots with a black line and shaded area represent a linear regression fit, and 95% confidence boundaries describing the relationship between time and threshold, and boxplots represent min, max, median, 25*th, *and 75*th *percentile*.

- *Means of communication:* Patient 1 interacted with the world by shifting attention (i.e., becoming quiet and still while listening). She demonstrated frustration through making fussy vocalizations and becoming more active.
- *Study optimization:* We opted to stimulate the left side because the patient had a broader range of motion, making leg flexion withdrawal feasible. Musical distraction techniques (familiar movie soundtrack) and parent interaction (soothing singing) were used while performing the test to help induce relaxation and reduce spurious leg movement. The patient was assessed while awake for all study time-points.
- *Interpretation:* Figure 1A shows baseline sensory threshold data collected across the duration of ASO drug treatment. Sensory thresholds remained stable throughout. The study site was relocated to a second site part-way through the study. Sensory threshold data collected at study site 1 and site 2 (by site-specific sensory physiologists) showed no clinical or statistically significant differences. Collectively, these data suggest reliability of the test across time and testing locations.

## 3-Year-Old Girl with Ataxia-Telangiectasia

Patient 2 underwent newborn screening, which showed a risk for severe combined immunodeficiency. At 7-months, she was diagnosed with ataxia-telangiectasia. At evaluation, at age 2.8 years, she presented with delays in speech and adaptive skills, and mild ataxia. Testing with the use of Vineland Adaptive Behavior Scales (Vineland-III) showed moderately low scores (3rd percentile) at baseline indicating that the patient was behind peers in development. Peabody Developmental Motor Scale (PDMS-2) scores were low for locomotion (2nd percentile), stationary motor (9th percentile) and object manipulation (9th percentile) at baseline.

- *Means of communication:* Patient 2 used non-verbal communication to indicate “yes” and “no,” vocalized to identify objects, and signs and gestures to indicate distress and frustration.
- *Study optimization:* The challenges faced were in ensuring stimulus-response specificity and patient engagement. We opted to (1) perform multiple trains of stimuli by applying each stimulus three times in three groups and documenting a positive response when 2/3 groups elicited a response; (2) alternate stimulus application location across the feet, so stimuli were “unexpected” (increasing attention and preventing habituation); (3) utilize relaxation techniques (play or eating); (4) protocol adaptations accordingly with developmental gains Verbal and non-verbal cues were considered positive responses, e.g., immediate changes in attention, leg withdrawal. The patient was assessed while awake for all study time-points.
- *Interpretation:* Figure 1B shows thresholds over time. Note that over the study time period, at age 3.8 years, verbal abilities improved, and she was converted from behavioral observation to adaptive self-report. Baseline sensory thresholds recorded on the day of drug dosing were consistent over time. As expected, the use of verbal-report as a means to communicate sensation provided a more sensitive compared to behavioral observation; both measures were stable over time.

## 18-Month-Old Girl with Epilepsy of Infancy with Migrating Focal Seizures

Patient 3 developed unrelenting seizures from day 4 of life onward. At age 4-months, she was diagnosed with a potassium channel mutation causing epilepsy of infancy with migrating focal seizures. At evaluation, the patient was preverbal with significant developmental delay and presented multiple seizures a day. Vineland Adaptive Behavior Scales (Villand-III) scores were low (<1st percentile) in all domains and indicated profound developmental delays across all domains at baseline. Motor evaluation showed severe gross hypotonia thoughout with minimal to no head control appreciated in any positioning; she required assistance for all mobility.

- *Means of communication:* Patient 3 had difficulty engaging in fundamental skills for attending to her environment and eliciting parent support for her needs. Parents were able to report behavious that suggested patient was hungry (i.e., lip smacking) or unsettled (i.e., groaning, startle-response).
- *Study optimization:* Being of toddler-age, the patient was often napping during the time window allocated for baseline data collection (i.e., hours prior to drug administration). We opted to take measurements when possible during both wakefulness and sleep states to ensure the measures were not floored.
- *Interpretation:* Sensory thresholds were stable over time but considerably lower during natural sleep compared to awake state (Figure 1C). Although her threshold during while sleep are lower they were consistent over time.

## Discussion

### Pros and Cons of Sensory Testing: Feasibility and Reliability

Various types of assessments can be performed to understand neurophysiologic function including QST (measurement of sensory thresholds), electrophysiologic testing (nerve fiber excitability), and recording of cortical sensory-evoked potentials. Here we have outlined individualized pragmatic approaches to QST in non-verbal research-participants undergoing treatment with novel ASOs as a component of clinical trial safety protocols. We have attempted to identify the most reliable sensory testing parameters given the patient age, state, disease presentation and cognitive ability. There are several advantages to our approach for use in these challenging patient populations.

One of the biggest challenges when working with pre/non-verbal populations lies in ensuring reliability of testing. The real-world data provided in this paper exemplifies how factors such as age and state can influence variability outcomes. We note that there is very little data available in this field, and as such, have largely drawn from our own experiences in this field. Where concerns over reliability of testing arise, it may be appropriate to complement sensory evaluation with more direct measures of nerve function. Alternative approaches include nerve conduction, electromyography, and cortical sensory-evoked potentials.

Electrophysiologic studies including nerve conduction velocity (NCV) and electromyography (EMG) have well-established interpretations in evaluation of peripheral nerve functioning. Conduction velocity in the upper extremities and lower extremities (ulnar, sural nerve stimulation) can be evaluated. Recording from one muscle supplied by the nerve distal to the site of stimulation is performed using surface electrodes, and/or a needle electrode. Slowing of nerve conduction velocity and reductions in amplitude usually indicate the presence of lesions affecting the axon of the peripheral nerve or loss of axons, respectively (43). Importantly, while NVC/EMG involves evaluation of nerve conduction in the largest and fastest myelinated fibers, it is insensitive to Að* and C-fiber dysfunction. Following proximal nerve injury or dysfunction, there is a time lag of several days before early changes are observed in NCV/EMG.

Cortical sensory-evoked potentials may provide additional objective information. Recording electrodes are placed over the scalp, and cutaneous stimuli are delivered. Cortical responses are analyzed in a similar approach to NCV/EMG. Laser, contact-heat, and intraepidermal electrical stimulation are the three main types of stimuli that can be used reliably. Thermal modalities primarily investigate small-diameter Að* fiber dysfunction (rather than C-fibers), and the central lesions involving the spinothalamic tract (44). Intraepidermal electrical stimulation preferentially activates large-diameter AB fibers rather than small-diameter Að* or C fibers (44). Both laser and contact-heat stimuli are associated with risk of burning at the cutaneous site of stimulation. As with NCV/EMG, these methods can cause discomfort so it is important that children have a sense of what to expect, and to implement these approaches on an as needs basis rather than for each time-point (which could be between 2 and 4+ times over a 24 h study period).

### Pros and Cons of Sensory Testing: Sensory and Motor Function Degradation

Challenges faced when designing a sensory testing study in a neurologically challenged population is in adapting to disease progression. Many genetic neurologic disorders are associated with neuropathies that may progress over time. Here, we opt to utilize the flexion withdrawal reflex as an outcome measure because of its primitive nature. However, for some patients, spasticity may develop and make it difficult to identify what is evoked vs. spontaneous limb movement. In this scenario, we recommend applying multiple stimuli and testing on multiple occasions.

We additionally provide a protocol that aims to generate objective and quantitative data. In pre- or non-verbal populations, experienced care givers may be used as a proxy for self-report although this approach is confounded by many challenges and biases. For example, patients may exhibit pain behaviors when they do not have pain and make it difficult to discern signs of pain, or observers may overestimate pain when unblinded to the application of a painful stimulus (45). However, when meeting the families for the first time we do ask parents/caregivers to provide us with insight of how patient communicate discomfort and we use these cues when assessing responses.

We show that our protocol can be used as a paragmatic approach to evaluate patient safety and drug toxicity during clinical trials were there is an potential risk for spinal toxicity. We additionally highlight the importance of interpreting the sensory testing results with other clinical examination assessments such as those performed by a neurologist and the physical therapist.

### Pragmatic Testing Considerations

We propose provisional recommendations for performing QST in children with neurologic disorders receiving early phase investigational intrathecal drug therapies based on our initial experience with N-of-1 trials.

#### General Protocol Composition

- Protocol design must balance obtaining necessary information while limiting session duration and exposure to noxious stimuli; e.g., adults might complete a QST battery lasting 1-h, whereas 5-year-olds might only tolerate a mechanical detection paradigm lasting 15-min.
- Documentation of experimental variables, e.g., patient positioning, sleep state, and distraction technique (Supplementary Figure 1).
- Documentation of degree of developmental disability across language, cognitive and motor domains (as appropriate).

#### Cognitive Level (Ability to Understand the Test)

- For neonates, young infants, or older children with developmental delay or neurologic regression who cannot provide self-report, mechanically evoked leg flexion withdrawal responses offer an indirect measure of sensory responsiveness. The outcome measure is the lowest mechanical stimulus intensity required to produce a response.
- Age-varying changes in mechanical threshold must be considered. The threshold required to evoke the reflex rapidly increases during the first 2 months of life and then changes very little up the first year (35, 36, 38).

#### Motor Function (Ability to Move in Response to a Stimulus)

- Patients with adequate understanding of the test but limited ability to verbalize the responses can be aided with alternative response cues, e.g., clicking a button or eye-blinking.
- Patients with movement disorders (e.g., spasticity or dystonia) presenting with stiff or restless legs make it difficult to evoke withdrawal responses. Optimizing positioning and distraction techniques facilitate relaxation and cooperation.

#### Cooperation (Ability to Withstand Multiple Repetitive Stimuli)

- Attention span and concentration affect the quality of the results by increasing data variability. Cooperation is enhanced in a quiet, calming environment and engaging through play or distraction (e.g., parental engagement or watching TV).

#### Baseline Measures

We recommend performing:

- A series of baseline evaluations on successive days that reflect changes in a patient's state and acknowledges that a degree of learned behavior will occur with longitudinal studies.
- Quality assurance tests where multiple assessors or study sites are involved.

## Conclusions

We have outlined specific considerations for developing individualized assessments for detecting changes in sensory processing in diverse patient groups and safety monitoring in N-of-1 trials of novel medications (Table 1). QST (either using self-report or nocifensive limb movements) is convenient for repeated bedside measurements. Changes due to mechanical injury, neurotoxicity, or reversible drug effect can be detected immediately, without the time delay of NCV/EMG. QST may therefore complement information obtained from the standard neurologic examination, electrophysiologic studies, skin biopsies, and imaging. QST has limitations and challenges, especially in non-verbal participants, as shown in the three cases discussed above. Future directions call for collaborative efforts to generate sensory datasets and share data registries in the pediatric field.

**Table 1:** Summary of general approaches for quantitative sensory testing in diverse groups.

**Patient features**	**Choice of QST paradigm**	**Output**
Typically developing or minor developmental delay with cognitive function at least 6 years old, + reasonably intact motor responses + able to provide verbal report	Full QST battery of 13 tests (as appropriate)	Self-report
Typically developing or minor developmental delay with cognitive function at least 6 years old, + reasonably intact motor responses + unable to provide verbal report	Full QST battery of 13 tests (as appropriate)	Self-report using adaptive communication aids, e.g., eye tracking or adaptive button
Significant developmental delay or cognitive function <6 years old + pre/non-verbal	Limited QST using 1 test, i.e., cutaneous mechanical stimulation of the foot	Gross motor behavior +/- facial expression responses, e.g., leg flexion withdrawal, grimace, vocalization
Significant developmental delay or cognitive function <6 years old + limited verbal response	Limited QST using 1 test, i.e., cutaneous mechanical stimulation of the foot	Individualized self-report +/- gross motor behavior, e.g., leg flexion withdrawal, play with giggle, head turning

## Data Availability Statement

The raw data supporting the conclusions of this article will be made available by the authors, without undue reservation.

## Ethics Statement

The studies involving human participants were reviewed and approved by Boston Children's Hospital Institutional Review Board. Written informed consent to participate in this study was provided by the participants' legal guardian/next of kin. Written informed consent was obtained from the minor(s)' legal guardian/next of kin for the publication of any potentially identifiable images or data included in this article.

## Author Contributions

CB, LC, and CD conceptualized and wrote the manuscript. LC, CD, CB, and TY critically revised the manuscript. All authors contributed to the article and approved the submitted version.

## Funding

This work is supported by the Sara Page Mayo Endowment for Pediatric Pain Research and Treatment, and by the Cardiovascular & Critical Care Cluster of Clinical Research Excellence at Boston Children's Hospital.

## Conflict of Interest

The authors declare that the research was conducted in the absence of any commercial or financial relationships that could be construed as a potential conflict of interest.

## Publisher's Note

All claims expressed in this article are solely those of the authors and do not necessarily represent those of their affiliated organizations, or those of the publisher, the editors and the reviewers. Any product that may be evaluated in this article, or claim that may be made by its manufacturer, is not guaranteed or endorsed by the publisher.
