# Supplementary material for: Modified Sensory Testing in Non-verbal Patients Receiving Novel Intrathecal Therapies for Neurological Disorders
Source: Front Neurol. 2022 Feb 10;13:664710. doi: 10.3389/fneur.2022.664710 (PMC8866183; doi:10.3389/fneur.2022.664710)
Supplement: Supplementary file 1 [file Data_Sheet_1.PDF]

Supplemental Material

ASO STUDY: CASE REPORT FORM

DATE: ______________ **Start time:** ______________ **Assessor:** ______________

PATIENT ID: ______________ **Finish time:** ______________ **Room:** ______________

STUDY SET-UP

1. Position (mark as appropriate)

a. Supine b. Seated c. Side lying left d. Side lying right

2. Sleep state (mark as appropriate; awake = eyes open; sleep = eyes closed)

a. Active awake (+ body movt, BM) b. Quiet awake (no BM) c. Sleep

3. Stimulation (mark as appropriate)

a. None b. Feeding/pacifier c. Distraction (TV/phone/music/parent interaction)

PART A: Tactile Protocol | LEFT SIDE STIMULATED (3x /hair) **Response: "/"**

TRIAL 1 | Hair # ->	1	2	3	4	5	6	7	8	9	10	11	12	13	14	15	16	17	18	19	20	Detection T1:
None																					
Toe																					
Ankle																					Nocicept. T1:
Knee																					
Hip																					

TRIAL 2	1	2	3	4	5	6	7	8	9	10	11	12	13	14	15	16	17	18	19	20	Detection T2:
None																					
Toe																					
Ankle																					Nocicept. T2:
Knee																					
Hip																					

TRIAL 3	1	2	3	4	5	6	7	8	9	10	11	12	13	14	15	16	17	18	19	20	Detection T3:
None																					
Toe																					
Ankle																					Nocicept. T3:
Knee																					
Hip																					

Figure S1: Case Report Form Template

Tactile Protocol is used evaluated cutaneous mechanical threshold using von Frey hair monofilaments. Detection is defined as stimulus intensity where any response is observed three times. Nociception threshold is defined as stimulus intensity where a knee- or hip flexion is observed three times.
